# “I’ll Worry About It Tomorrow” – Fostering Emotion Regulation Skills to Overcome Procrastination

**DOI:** 10.3389/fpsyg.2022.780675

**Published:** 2022-03-22

**Authors:** Laura Schuenemann, Viviane Scherenberg, Maria von Salisch, Marcus Eckert

**Affiliations:** ^1^Department of Psychology, Leuphana University of Lüneburg, Lüneburg, Germany; ^2^Department of Psychology and Education, Apollon University of Applied Sciences, Bremen, Germany; ^3^Institute of Psychology, Leuphana University of Lüneburg, Lüneburg, Germany

**Keywords:** overcoming procrastination, emotion regulation, emotion regulation skills training, e-mental health intervention, procrastination, stress intervention

## Abstract

Procrastination remains an omnipresent phenomenon impeding especially students’ academic performance and well-being. Preliminary findings suggest that procrastination emerges due to dysfunctional emotion regulation efforts to regulate aversive emotions. This study’s objective was to clarify whether the enhancement of general adaptive emotion regulation skills reduces subsequent procrastination. For the purpose of this study, data from a two-armed randomized controlled trial (RCT) with (*N* = 148) university students, comprising an active intervention (IG) and a passive wait-list control (WLC) group, was collected. Participants of the intervention group were provided with an online emotion regulation training over a period of 9 weeks. The results showed that the enhancement of general emotion regulation skills significantly reduced subsequent procrastination behavior within the IG as compared to the untreated WLC. Moreover, subsequent mediation analyses revealed that the reduction of procrastination was significantly mediated by the increase in general ER skills. The present results suggest that trainings which enhance general ER skills are an appropriate measure to reduce procrastination behavior among university students. The practical value of ER training interventions, particularly for student populations, is discussed.

## Introduction

In recent years, increasing research has addressed the question, why individuals rather watch cat videos online (Myrick, 2015), and students prefer to watch TV or sleep instead of executing their mandatory tasks (Pychyl et al., 2000). Procrastination remains a widespread phenomenon, whose existence, according to Steel (2007), can be traced back to 400 BC. Today, more than two thousand years later, we are still in the process of understanding the phenomenon. Approximately 15–20% of the total adult population suffers from chronic procrastination (Harriott and Ferrari, 1996; Ferrari et al., 2007; Steel, 2007), and over 95% wishes to minimize their procrastination behavior (O’Brien, 2002). Among student populations, the prevalence is even higher. Estimates show that 80–95% of students engage in occasional procrastination (Steel, 2007), and approximately 50% suffer from chronic, detrimental procrastination behavior (Day et al., 2000; He, 2017). Although some researchers argue that procrastination does not only comprise negative effects (Chu and Choi, 2005), most research has put forward that procrastination highly impedes indicators of success and performance (Steel, 2007; Gareau et al., 2018), individuals’ mental (Strongman and Burt, 2000; van Eerde, 2003; Sirois and Pychyl, 2013) and even physical health (Sirois, 2015). With regard to these figures, procrastination remains a well-discussed topic in psychological research and must be targeted in order to enhance individuals’ and especially students’ performance, health and overall well-being. The key question that needs to be provided with empirical evidence is how the phenomenon can be overcome and the resulting issues diminished.

In current literature, procrastination is generally understood as “to postpone completing a task from the present or near future to a more distant future” (Gautam et al., 2019, p. 1). The term procrastination originates from the Latin words pro, meaning “in favor of or forward,” and crastinus, meaning “of tomorrow” (Steel, 2007, p. 66). Procrastination encompasses a voluntary and irrational delay of an intended action (Ellis and Knaus, 1977; Burka and Yuen, 1983; Akerlof, 1991; Steel, 2007; Sirois and Pychyl, 2013), despite the awareness that one will not maximize one’s gains (e.g., personal interests, preferences, material, and psychological goals; Steel, 2007), instead harm one’s future-self by this course of action (Sirois and Pychyl, 2013). By procrastinating, we expect “that tomorrow will be different […] that we will be different tomorrow” (Sirois and Pychyl, 2013, p. 116). However, procrastinators deliberately and irrationally postpone an intended action although anticipating being worse off after, due to the negative consequences caused by the delay (Steel, 2007; Klingsieck, 2013; Sirois and Pychyl, 2013).

### Procrastination as a Dysfunctional Emotion Regulation Strategy

In previous literature the ability to regulate emotions adaptively and properly in explaining the general tendency to procrastinate has been stressed (e.g., Tice and Baumeister, 1997; Tice and Bratslavsky, 2000; Sirois and Pychyl, 2013; Eckert et al., 2016). Several researchers have suggested that procrastination is rooted in the application of a dysfunctional emotion regulation strategy (Sirois and Pychyl, 2013, 2016; Eckert et al., 2016) resulting from emotional misregulation (Sirois and Pychyl, 2013). Moreover, it has been highlighted that aversive emotions represent the key antecedents of procrastination (Steel, 2007; Eckert et al., 2016; Pollack and Herres, 2020).

Accordingly, investigating how the enhancement of adaptive emotion regulation skills affects subsequent procrastination behavior could help improving existing and developing novel interventions to reduce procrastination and its associated adverse effects. Thus, this study will first theoretically and subsequently experimentally demonstrate how the enhancement of general adaptive emotion regulation skills could aid in overcoming the illustrated phenomenon.

Diverse intervention programs to reduce procrastination have been developed and evaluated (van Eerde and Klingsieck, 2018), including interventions based on Cognitive Behavioral Therapy (Toker and Avci, 2015; Eckert et al., 2018; Rozental et al., 2018), based on self-regulation (Grunschel et al., 2018) and emotion regulation strategies (Eckert et al., 2016), and interventions building on Acceptance and Commitment Therapy (ACT) and mindfulness (Dionne, 2016). Evidently, the majority of the existing intervention programs include emotion regulation processes and address them in a direct or indirect manner. To improve and sharpen the existing interventions, it is necessary to understand how the enhancement and acquisition of adaptive emotion regulation skills affects subsequent procrastination.

### Emotion Regulation

Emotion regulation (ER) is defined as a multifaceted and broad construct including all “extrinsic and intrinsic processes responsible for monitoring, evaluating, and modifying emotional reactions, especially their intensive and temporal features, to accomplish one’s goals” (Gross, 2007, p. 251). Preliminary findings suggest that individuals engage in ER processes to enhance pleasure and prevent pain (Larsen, 2000; Gross, 2014). The most scientifically examined model, the Process Model of Emotion Regulation by Gross (1998, 2014), portrays the different steps of the emotion-generative process. It distinguishes between five strategies that individuals can engage in order to regulate their emotions: (1) situation selection, (2) situation modification, (3) attention deployment, (4) cognitive change, and (5) response modulation. These strategies each target a different emotion-generative step and can be addressed to influence the emotional dynamics of the emotional response (Thompson, 1990), including the intensity, duration, magnitude, and offset of behavioral, physiological, and experiential responses (Gross, 1998). Engaging in healthy ER processes, thereby, refers to the ability to alter how intensely, how long, and how fast one feels one’s emotion, rather than changing the valence of one’s emotion (Gross, 2007). Accordingly, ER as a process includes the upregulation and downregulation of the extent or duration of one’s emotional responses by applying several emotion-focused strategies (Gross, 2014; Sirois and Pychyl, 2016). These ER strategies are rarely maladaptive or ideal by definition, instead, the strategies must be considered in the context of a person’s goal for a certain situation (Gross, 2007). Regarding procrastination, the engagement in certain ER strategies can be dysfunctional, if they involve the downregulation of aversive emotions at the cost of one’s previous formed intentions and goals. According to Sirois and Pychyl (2016) procrastination can be understood as a self-regulatory failure that arises due to preceding aversive emotions, such as frustration, boredom, negative affect, anxiety, and worry (Blunt and Pychyl, 2000) resulting from the exposure to unpleasant and aversively perceived tasks (Lay, 1990). Thus, by procrastinating, the engagement in the voluntarily delay of unpleasant but mandatory tasks, individuals manage to avoid the experience of the aversive emotions in short-term, albeit fail to achieve their long-term goals (Sirois and Pychyl, 2016). Respectively, aversive affective states have been proposed to represent the key antecedents of procrastination (Eckert et al., 2016; Pollack and Herres, 2020). Accordingly, when considering procrastination as a self-regulatory failure resulting from emotional misregulation, it follows logically that the acquisition of ER skills to cope with aversive emotions adaptively may aid in preventing procrastination.

### Emotion Regulation Skills

As depicted in the previous section, ER represents an exceedingly dynamic process (Ochsner and Gross, 2007). Apart from the outlined ER strategies, ER can be understood as skills operating in the ER process. These skills can be conceptualized as an element of the ER process and are fundamental for adaptive coping with aversive emotional states, and therefore, the engagement in healthy ER processes (Berking et al., 2008, 2012). To provide a conceptual framework for the relevant ER skills involved in adaptive ER processes and adaptive coping Berking and Whitley (2014a) have put forward the Adaptive Coping with Emotions Model (ACE; Berking and Whitley, 2014b).

According to the ACE model, adaptive ER is defined as the situation-adapted interaction of the ER skills to: (1) be aware of one’s emotions, as it represents a prerequisite for conscious emotion regulation, (2) identify and correctly label one’s emotions, as it facilitates the management of the experienced emotions, (3) correctly interpret emotions related to bodily sensations, as it aids in avoiding misinterpretations that can fuel psychological or psychosomatic symptoms, (4) understand the cause of emotions, as it permits the discovery of opportunities of change, (5) adaptively modify aversive emotions to enhance one’s emotional state and build self-efficacy, (6) accept one’s emotions, as the non-acceptance of one’s emotions fuels the emergence of further negative emotions, (7) be resilient (tolerate and accept aversive emotions), as dysfunctional control efforts contribute toward the maintenance of negative emotions and emotions are regulated by brain regions that elude voluntary control efforts (Berking, 2010), (8) emotionally support oneself in distressing situations to avoid impulsive behavioral mood-repair responses, and (9) confront distressing situations eliciting negative emotions to acquire effective ER competencies and build resilience (Berking and Znoj, 2008).

In view of the ACE model, adaptive ER skills enable adaptive coping with aversive emotions. In line with previous research (Eckert et al., 2016; Sirois and Pychyl, 2016), this study considers procrastination as a dysfunctional ER strategy and precisely a maladaptive coping advance, concerning the dysfunctional regulation of aversive emotions. Therefore, deficits in ER skills can be considered as the origin of the misregulative emotional nature of procrastination.

Previous research indicates that general ER skills can be acquired and fostered (Berking et al., 2008; Berking and Lukas, 2015; Eckert and Tarnowski, 2017). As the significance of adaptive ER skills to preserve mental and physical health and prevent psychopathological symptoms has been shown by numerous studies (Wirtz et al., 2013; Berking et al., 2014; Trindade et al., 2017; Cloitre et al., 2019), it is proposed that deficits in ER skills must be altered to allow adaptive coping with the preceding aversive emotions, and consequently prevent procrastination, the resulting distress, and the adverse health consequences.

### Objective and Hypotheses

In light of the given evidence, it is expected that the enhancement of general ER skills prevents subsequent procrastination behavior. A pioneering study by Eckert et al. (2016) was the first to cross-sectionally and longitudinally demonstrate that ER skills affect subsequent procrastination behavior. Moreover, within a 2-week RCT they showed that the enhancement of task-related ER skills significantly reduced procrastination among university students. Prior research, therefore, is limited to the enhancement of ER skills cued by tasks. Despite the interest, the enhancement of task-unrelated general ER skills aimed at reducing procrastination has not been studied yet. By further investigating this research gap, it is intended to expand current scientific literature on the interrelationship of procrastination and ER. Most importantly, it is intended to provide empirically founded knowledge on feasible interventions targeting the reduction of procrastination and its associated detrimental effects. Given that the present intervention is conducted on a university student sample, with respect to educational purposes, this research aims at providing a contribution toward a successful intervention program targeting academic procrastination to enhance performance and well-being among students.

The present study investigates the effects of an online emotion regulation skills training on subsequent procrastination behavior among university students. First, we assume that the intervention increases general ER skills (hypothesis 1) compared to a waiting-list control group (WLC). In line with hypothesis 2, this study postulates that the present ER intervention results in a significant reduction of the dependent variable, procrastination, among the intervention group (IG) from baseline (t1) to post-measurement (t2) as compared to WLC. In hypothesis 3, it is proposed that the expected treatment effect (the reduction of procrastination at t2) will be attributed to the overall acquisition of adaptive ER skills and will be tested using a mediation analysis.

***H1* (Treatment Efficacy)**: Participants of the intervention group (IG) will significantly increase general ER skill from t1 to t2 in comparison to participants of the WLC.

***H2* (Treatment Efficacy)**: Participants of the intervention group (IG) will significantly procrastinate less from t1 to t2 in comparison to participants of the WLC.

***H3* (Mediation Analysis)**: The reduction of participants’ procrastination behavior at t2 will be significantly mediated by the overall increase in the participants’ general ER skills.

## Materials and Methods

### Research Design

The research design consisted of a two-armed randomized controlled trial (RCT) comprising an active intervention group (IG), provided with a 9-week online ER training enhancing general adaptive ER skills, and a passive wait-list control group (WLC) without the administration of a placebo. The mixed-subject design measured the between factor *treatment* with two levels (intervention- vs. wait-list control group) and the within factor *time* with two levels [baseline (t1) vs. post-measurement (t2)]. Procrastination and ER were assessed prior to the treatment (t1), and immediately after the 9-week intervention period (t2). Based on prior findings (Eckert et al., 2016; Eckert and Tarnowski, 2017), the current intervention was expected to have at least a medium-sized effect on procrastination. According to the power analysis a total sample size of 138 participants was required to detect a medium effect size of η^2^ = 0.06 with 95% power (α = 0.05). Since a certain drop-out rate at post-measurement (t2) was to be expected, it was oversampled about 20% at baseline measurement (t1). Thus, the data collection at t1 was stopped once about 170 participants had registered for the study. All procedures were in line with the Institutional Review Board at Leuphana University of Lüneburg in Lüneburg, Germany.

### Participants and Procedure

The sample of the present study mainly consisted of university students, as previous research indicated that approximately 50% of students report serious procrastination issues (Day et al., 2000). The participants were recruited from the Leuphana University of Lüneburg (Germany) via the university intern research participation platform Sona Systems^1^ and other social networks between April 27th and May 6th, 2020. The data collection of the pre-measurement occurred during the recruitment period. The acquisition of the post-measurement data was conducted between June 26th and July 15th. Participants of the Psychology Department of the Leuphana University of Lüneburg were compensated with two credits as an incentive for their participation in the study. Students from all other departments and external students did not earn any further compensation in addition to the online training received free of charge.

All individuals that completed the baseline online survey (t1) and provided informed consent were included in the study. The presented questionnaires were in German and could be completed on a computer, tablet, or smartphone. The processing time was scheduled for approximately 15 min. The subjects were instructed to provide their email address and name. Shortly after, they received a personalized email with a link that forwarded them to the pre-measurement survey. Simultaneously, the integrated random generator randomized them toward IG or WLC and assigned them an internal serial number, enabling their identification at t2. The participants were asked to complete an online questionnaire measuring their baseline ER skills, procrastination scores, and socio-demographic data, comprising items regarding age, gender, and course of studies. Subsequently, the participants received an automated personalized email confirming their group allocation (IG vs. WLC). The IG directly received the online ER training “Stark im Stress” (*SIS*)^2^ for 9 weeks. The WLC received the training right after the intervention period ended and the successful completion of the post-measurement questionnaire. After the intervention period of 9 weeks, all participants received a link via a personalized email, inviting them to complete a post-measurement questionnaire, consisting of the same procrastination and ER measures. Data protection was guaranteed at all times, as the analyses were carried out anonymously.

**FIGURE 1:**
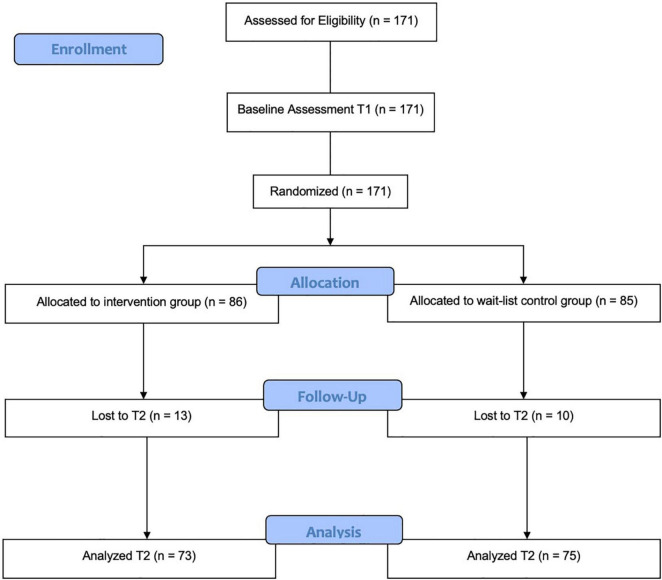
CONSORT flow diagram.

At baseline measurement (t1), the sample consisted of *N* = 171 participants, whereof 85 participants were randomly assigned to the WLC and 86 to the IG (see Figure 1 for the complete CONSORT flow diagram). As 23 (13.5%) cases, 10 from the WLC and 13 from the IG, had to be excluded due to unfinished questionnaires or drop-outs at post-measurement, the valid final sample size amounted to *n* = 148 participants, consisting of 111 women (75.0%), 36 men (24.3%), and 1 diverse gendered participant (0.7%). As some statistical tests regarding the sample characteristics could only be performed with two groups, the participant specifying diverse gender had to be excluded from those statistical analyses where gender constituted a factor. This measure should not be considered discriminatory under any circumstances. The average age of the participants was 22.6 years (*SD* = 3.7; men: *M* = 23.8, *SD* = 5.0; women: *M* = 22.2, *SD* = 3.0) with a range of 18–43 years. Within the final sample, 143 (96.6%) participants were currently enrolled as university students. The remaining five (3.4%) participants reported being employed. Of the 148 participants, 72 (48.6%) of the students indicated psychology as their course of studies, 40 (27.0%) law and economics, 14 (9.5%) participants declared the STEM fields as their major, 13 (8.8%) cultural, media and communication sciences and four (2.7%) social sciences. A Fisher’s exact test indicated no significant allocation difference regarding gender and course of studies (*p* = 0.608). 75 participants were allocated toward the WLC and 73 toward the IG. A Mann–Whitney *U* test indicated that the participants were allocated to the IG and WLC evenly regarding age (*p* > 0.05). A Pearson chi-square test revealed a significant distribution difference regarding gender between treatments [χ^2^(1) = 5.084, *p* < 0.05], and a Fisher’s exact test showed a significant difference regarding course of studies between the groups (*p* < 0.05).

### Intervention

The participants were provided with the German online ER training “Stark im Stress” (Eckert and Tarnowski, 2017), consisting of three main modules. In line with previous findings (Berking et al., 2012; Eckert et al., 2016), each module addresses different ER skills, particularly focusing on the ER skills to tolerate, accept, and modify aversive emotions. The first module targets the enhancement of personal resources through the instruction and practice of mindfulness (Hill and Updegraff, 2012) and relaxation exercises (Berking and Whitley, 2014a), the establishment of positive emotions (Dalebroux et al., 2008; Alladin and Amundson, 2016), and regeneration. In the first session, the participants learn to evoke positive emotions by imaginations. In the following session, the participants receive an audio file supporting them in their practice of mindfulness and relaxation. The file includes a mindfulness exercise, which invites the participants to focus their attention on their breath. Additionally, it contains a guided exercise to practice a short version of progressive muscle relaxation, which requires approximately 15 min to complete (Dolbier and Rush, 2012). In session three, the participants are instructed to plan regeneration breaks and are taught how to cope with potential obstacles.

The second module encourages the participants to tolerate and accept negative emotions by applying behavioral activation techniques (Chu et al., 2009), self-esteem strategies (Berking and Whitley, 2014a; Kolubinski et al., 2018), and benefit-finding (Bower and Segerstrom, 2004). Therefore, in session four, the participants plan pleasant activities, which function as a prevention of negative moods. Session five focuses on benefit finding strategies. In session six, the participants are instructed to remember personal successes, their personal competencies, appreciations and self-cherishing in order to increase their self-esteem (Berking and Whitley, 2014a).

The third module aims at the modification of negative emotions by cognitive (Berking and Whitley, 2014a; Morris et al., 2015) and embodiment techniques (Tschacher and Pfammatter, 2016). Therefore, in the seventh session the participants are invited to apply embodiment techniques. In session eight the participants are requested to identify typical dysfunctional cognitions causing stress and learn how to modify them. In the last session the participants are instructed to integrate the strategies that personally work best for them in order to be able to adaptively cope with future aversive emotions.

Each module consisted of three 90-min units, comprising online educational video lessons and the accompanying support of a booklet. An additional app provided daily text message-based reminders to engage in quick ER exercises as the benefit of text messages has been shown to increase training adherence significantly (Eckert and Tarnowski, 2017), especially among procrastinators (Eckert et al., 2016). Message-based reminders may facilitate the initiation of action and apply the foot-in-the-door technique (Eckert et al., 2018).

### Measurements

#### Procrastination

Procrastination was measured using the German short version of the General Procrastination Scale (*GPS*; Lay, 1986; German version: *GPS-K*; Klingsieck and Fries, 2012). Due to the lack of psychometric validity, Klingsieck and Fries (2012) factor-analytically revised the original GPS scale. The GPS-K is a self-report instrument including nine items that are each rated on a 4-point Likert-type response scale (1 = *extremely uncharacteristic* to 4 = *extremely characteristic*). Four of the nine items are inversed. A sample item is “I often find myself performing tasks that I had intended to do days before” (Lay, 1986). To compute a total scale score, all ratings are summarized and divided by the number of items (9). Higher values indicate higher self-reported procrastination behavior. In the present study, the internal consistency of the GPS-K was excellent (α_*t*1_ = 0.90). The corrected item-total correlation (r_*it*_ = 0.63–0.76) and the test-retest reliability (r_*tt*_ = 0.81) indicated acceptable values.

#### Emotion Regulation

Emotion regulation skills were assessed using the Emotion Regulation Skills Questionnaire (*ERSQ*; German version: Berking and Znoj, 2008). The ERSQ is a self-report instrument that includes 27 items and utilizes a 5-point Likert-type scale (1 = *not at all* to 5 = *almost always*) to assess adaptive emotion regulation skills (Berking and Znoj, 2008). However, in the present study, a four-point scaling was used, as also indicated in the validation paper by Berking and Znoj (2008). The ERSQ assesses nine specific ER skills (awareness, sensations, clarity, understanding, acceptance of aversive emotions, resilience, self-support in distressing situations, readiness to confront distressing situations, and modification) with subscales composed of three items each. The items are preceded by the stem, “Last week ….” Sample items include: “I paid attention to my feelings” (awareness); “my physical sensations were a good indication of how I was feeling” (sensations); “I was clear about what emotions I was experiencing” (clarity); “I was aware of why I felt the way I felt” (understanding); “I accepted my emotions” (acceptance of aversive emotions); “I felt I could cope with even intense negative feelings” (resilience); “I did what I had planned, even if it made me feel uncomfortable or anxious” (readiness to confront distressing situations); and “I was able to influence my negative feelings” (modification). Emotion regulation was successfully assessed by averaging all items to compute a total score (Berking and Znoj, 2008). In the present study the internal consistency of the total ERSQ scale score was excellent (α_*t*1_ = 0.91). The subscales’ corrected item-total correlation achieved acceptable values (r_*it*_ = 0.37–0.78), equally the respective internal consistencies (α = 0.63–0.87). The test-retest reliability for the total ERSQ scale score was r_*tt*_ = 0.55.

### Statistical Analysis

The descriptive and inferential statistical analyses of the data were carried out, after the transformation of the protocols from SoSci Survey (Leiner, 2020) on the basis of the corresponding data matrix. All descriptive and inferential statistical analyses were conducted using IBM SPSS^®^ 22.0 for Mac OSX (IBM Corp., 2013).

#### Treatment Efficacy

For the analysis of hypothesis 1, thus, to examine whether general ER skills were increased significantly among the treated IG as compared to the untreated WLC, the changes in ERSQ scores over time due to the treatment (within group effect) and the differences between the conditions at t2 (between group effect) were tested using a MANOVA. To test hypothesis 2 and to assess the treatment efficacy, a two-way (2 × 2) mixed rmANOVA was calculated, with *time* (t1 vs. t2) as the within-subjects factor, *treatment* (IG vs. WLC) as the between-subjects factor and *procrastination* as the dependent variable. The advantage of this method is that it allows the analysis of interactions of several factors on the dependent variable, besides the analysis of the main effect. To answer hypothesis 2 the outcome values of the dependent variable (GPS-K mean scores) of the IG group were compared with those of the WLC group. In addition, effect sizes (Cohen’s *d*) were calculated to estimate the within effect (t2 – t1 for each group) and the between effect (IG vs. WLC at t2) of the training. Accordingly, follow-up independent sample *t*-tests were used to analyze whether the two groups significantly differed in procrastination scores at baseline and at post-measurement. Follow-up paired sample *t*-tests were used to test the reduction from t1 to t2 in procrastination scores for significance in both groups.

#### Mediation Analysis

For the analysis of hypothesis 3, thus, to investigate whether the expected treatment effect could be attributed toward the enhancement of general ER skills, a mediation analysis, according to Hayes (2018), was performed, as the causal steps approach by Baron and Kenny (1986) has been scientifically criticized due to low statistical power and the non-direct testing of one indirect effect (Preacher and Hayes, 2004; Hayes, 2009; Rungtusanatham et al., 2014). Therefore, to validly test the mediation effect and draw conclusions concerning the hypotheses, this study relied on the significance of indirect effects (the effect of the mediator). Indirect effects were estimated by calculating a point-estimate for the product of a × b and interval-estimates (*CI*; confidence interval; Hayes, 2018). The mediation analysis was conducted using PROCESS macro (2.16.3) by Hayes (2018), which applies linear regression to detect unstandardized path coefficients for total, direct, and indirect effects by making use of ordinary least squares. The bootstrap-based test provides the advantage of requiring the smallest sample size, providing the best power (Fritz and MacKinnon, 2007), and not requiring normally shaped sampling distributions (Preacher and Hayes, 2004).

## Results

### Treatment Efficacy

To examine whether the ER intervention significantly increased general ER skills among the IG as compared to the WLC, a MANOVA was calculated. Accordingly, a preliminary calculation of the difference in ERSQ scores (t2–t1) was carried out and subsequently tested with a one-way MANOVA. In line with hypothesis 1, the MANOVA indicated a significant increase for the total scale score in general ER skills among the IG from pre- to post-measurement in comparison to the WLC (*F_1,146_* = 26.987, *p* < 0.001, partial η^2^ = 0.16). Table 1 depicts the means and standard deviations of the ERSQ score for the baseline (t1) and post-measurement (t2) for both groups as well as the test statistics and effect sizes. Regarding the within group effect sizes, the increase in general ER skills from t1 to t2 was considered large within the IG (*d*_*within*_ = 0.9) and very small within the WLC (*d*_*within*_ = 0.06). The size of the between group effect at post-measurement was medium (*d*_*between*_ = 0.48).

**TABLE 1:** Means, standard deviations, and test statistics of the procrastination scores (GPS-K) and ER skills scores (ERSQ) for the intervention (IG) and the wait-list control group (WLC).

	WLC (*n* = 75)		IG (*n* = 73)		Test statistics		
Scale	*M*t1 (*SD*)	*M*t2 (*SD*)	*M*t1 (*SD*)	*M*t2 (*SD*)	*F*(1,146)	*p*	η^2^
GPS-K	2.85 (0.66)	2.70 (0.67)	2.81 (0.63)	2.43 (0.53)	14.498	<0.001***	0.09
ERSQ	3.03 (0.45)	3.06 (0.49)	2.89 (0.48)	3.26 (0.33)	26.987	<0.001***	0.16

N = 148. M, Mean; SD, Standard deviation; η^2^, Eta-square; p, p-value; GPS-K, General Procrastination Scale; ERSQ, Emotion Regulation Skills Questionnaire. ***p < 0.001.

To test hypothesis 2, and thereby examine the changes in procrastination scores from t1 to t2 among both groups, time as the within-subjects factor and treatment as the between-subjects factor were included in the two-way (2 × 2) mixed rmANOVA. Both the main effect for the factor time (*F_1,146_* = 72.868, *p* < 0.001, partial η^2^ = 0.33) as well as the interaction term “time × treatment” (*F_1,146_* = 14.498, *p* < 0.001, partial η^2^ = 0.09) were significant. The main effect of the within-subjects factor time had to be interpreted individually for each group and was therefore tested using follow-up paired sample *t*-tests. The results of the analysis comprising the means, standard deviations, and test statistics regarding both time and treatment are displayed in Table 1.

**FIGURE 2:**
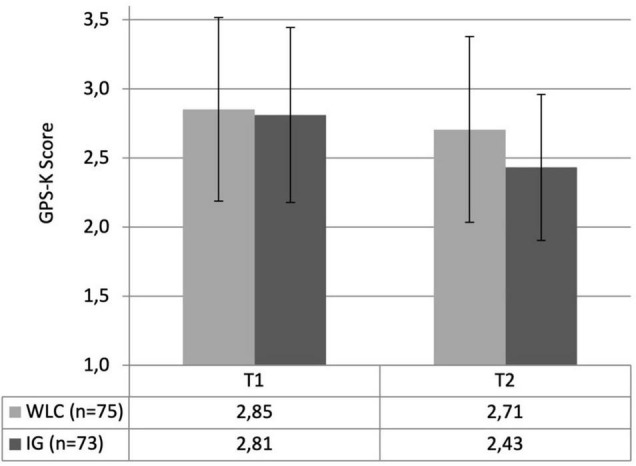
Changes in procrastination scores (GPS-K) from pre- (t1) to post-measurement (t2) in relation to the treatment factor (WLC vs. IG).

In both groups the procrastination mean scores (GPS-K) decreased over time. The results of the paired sample *t*-tests indicated that the reduction of procrastination from t1 to t2 was significant in both groups, with *t*_74_ = 3.593, *p* = 0.001 within the WLC and *t*_72_ = 8.172, *p* < 0.001 within the IG. The corresponding effect sizes for the reduction in procrastination were *d*_*within*_ = 0.41 within the WLC and *d*_*within*_ = 0.96 within the IG. To determine the actual treatment effect of the training, the effect of the IG was adjusted for the effect of the WLC (by subtracting the effect of the WLC) and expressed as a relativized effect. The relativized effect resulted in a total within effect of *d* = 0.55. Figure 2 displays the reduction of procrastination behavior.

Considering the significant interaction effect, for the between-subjects effect, subsequent pairwise comparisons between the two groups at both measurement points were carried out. The independent sample *t*-test indicated that there was no significant difference in procrastination scores between the groups at baseline measurement, *t*_146_ = 0.381, *p* = 0.704, but that the IG and WLC significantly differed in procrastination scores at post-measurement, *t*_140_ = 2.764, *p* = 0.006, *d*_*between*_ = 0.45. The between effect has a small magnitude and therefore indicated a small difference in the GPS-K scores between the IG and WLC at post-measurement.

### Analysis of Mediation

In this section, a mediation analysis, in line with hypothesis 3, is reported to test the mediating influence of general ER skills from the treatment variable on the changes in procrastination scores at t2. The mediation analysis was performed based on model 4, according to Hayes (2018). For the analysis, the treatment condition (IG vs. WLC) was used as the independent variable, procrastination scores at t1 as the covariate, the differences in ERSQ scores between t2 and t1 (ERSQ_*t*2_ – ERSQ_*t*1_ = ΔERSQ) as the mediator variable, and procrastination at t2 as the dependent variable.

**FIGURE 3:**
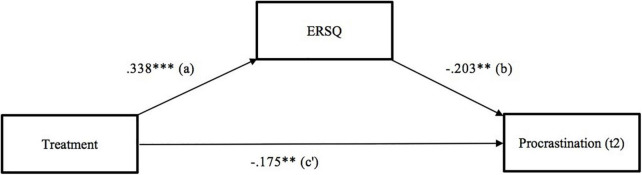
Results of the mediation analysis with ΔERSQ as a mediator. Path analysis with unstandardized coefficients (β), according to Hayes (2018). All paths indicate significant effects. *N* = 148. **p < 0.01; ***p < 0.001. a = path a, b = path b, c′ = path c′.

The path coefficient for the direct effect (c′) controlling the mediator ERSQ_t2-t1 resulted in, β = −0.175, *p* = 0.004, *95%-CI* [−0.294, −0.056], indicating a significant result. Both the a and b path displayed significant results (a: β = 0.338, *p* < 0.001, *95%-CI* [0.210, 0.466]; b: β = −0.203, *p* = 0.004, *95%-CI* [−0.294, −0.056]). The total effect (c), which represents a linear additive model consisting of the direct (c′) and indirect effect (the product of a × b), showed a small significant negative effect, β = −0.243, *p* < 0.001, *95%-CI* [−0.355, −0.132]. To validly test hypothesis 3, following contemporary scientific recommendations (Memon et al., 2018), this study relied on a 95% bias-corrected confidence interval based on 5,000 bootstrap samples, which revealed a significant indirect effect (β = −0.068, *95%-CI* [−0.125, −0.023]). Regarding the significant indirect effect (see Preacher and Hayes, 2004, for more details), the results indicated a significant mediation effect from treatment (IG vs. WLC) on procrastination scores at t2 via the increase in overall ER skills. Figure 3 shows the respective mediation analysis with path significances according to Hayes (2018).

## Discussion

The present study first-time investigated whether the enhancement of general, task-unrelated ER skills through an online ER training significantly reduces subsequent procrastination behavior among university students. To examine the expected treatment effect adequately, this study was designed as a randomized controlled trial with two independent conditions, an intervention- (IG) and a wait-list control group (WLC) and two measurement periods consisting of a baseline (t1) and a post-measurement (t2). Consistent with the main hypotheses, the results revealed that the applied ER training increased general ER skills among the IG significantly as compared to the WLC (H1) and reduced in a student sample the general tendency to procrastinate among the IG, as compared to the untreated WLC with a medium-sized effect (H2). In line with the third hypothesis, the results showed that the reduction of procrastination was mediated by the increase in general ER skills. Thus, it can be assumed that enhancing and fostering ER skills possesses the potential to reduce the postponement of intended tasks.

Procrastination represents a widespread phenomenon affecting almost all age groups (Ferrari et al., 2007; Steel, 2007) and especially up to 95% of the student population (Ellis and Knaus, 1977; Steel, 2007), potentially causing several mental health issues (e.g., Ferrari, 1991; Strongman and Burt, 2000), and negatively affecting indicators of performance and success (e.g., Gareau et al., 2018). Therefore, the objective of this study constituted the prevention of procrastination and its harmful consequences. Building on the assumption that preceding aversive emotions trigger subsequent procrastination behavior through the application of a dysfunctional ER strategy that enables the short-term avoidance of the antecedent aversive emotions (short-term mood repair; Sirois and Pychyl, 2013, 2016), the present research proposed that fostering general adaptive ER skills prevents the usage of procrastination as a dysfunctional ER strategy. In line with the hypotheses and preliminary findings of Eckert et al. (2016), the present results showed that the training of ER strategies enhancing general ER skills significantly reduced subsequent procrastination behavior from pre- to post-measurement among the IG, as compared to the untreated WLC, with a relativized medium-sized effect. From a theoretical point of view, the results provide empirical evidence for the prediction that the enhancement of adaptive ER skills reduces the employment of the dysfunctional ER strategy procrastination. When adaptive ER skills are acquired, they facilitate adaptive coping with preceding aversive emotions regarding a specific task.

Previous research by Eckert et al. (2016) has already highlighted that task-related ER strategies can prevent procrastination. Within their research, ER strategies were directly applied to overcome the aversive emotions associated with an unpleasant task. Adding onto a scarce body of scientific literature, the present study revealed that fostering general task-unrelated ER skills also exerts an influence on procrastination. The results, thus, provided empirical evidence for the indirect effect that general ER skills exert on procrastination. This suggests that acquiring new and enhancing existing ER skills, even in a relatively short intervention period of 9 weeks, can result in significant reductions in procrastination behavior among students. Therefore, online ER training interventions should be considered an effective measure for educational institutions to reduce procrastination and the associated adverse health outcomes. Consistent with the findings of Eckert et al. (2016), the between effect, indicating the difference of procrastination scores between the IG and the WLC at t2, displayed a small to medium effect size (*d*_*between*_ = 0.45). Similarly, a study by Rozental et al. (2015) showed that online-based self-help cognitive-behavior therapy resulted in moderate changes in procrastination scores, especially among the intervention group with the guidance of a therapist, but also among the unguided self-help group. Taking into consideration the various factors that can cause procrastination (Walsh and Ugumba-Agwunobi, 2002; van Eerde, 2003; Steel, 2007; Eckert et al., 2016), rather small effects were expected upfront. Nonetheless, given the detrimental consequences of procrastination, even smaller effects should not be underestimated, as marginal behavioral changes can exert strong influences on subsequent performance and well-being. In addition, practical relevance must always be interpreted in light of costs and expenditure. Given the cost-efficient online-based format of the applied ER training, the present intervention is highly suited for the practical context. Especially in view of the large proportion of students procrastinating on a regular basis (up to 95%; Steel, 2007), resulting in lower assignment- and course grades (Steel, 2007; Gareau et al., 2018), the present findings indicate that students would significantly procrastinate less through the implementation of ER trainings and accordingly enhance their procrastination-related impaired performance.

The results of the mediation analysis supported the assumption that the overall increase in ER skills mediates the relationship between group allocation and procrastination scores at post-measurement. Thus, the total increase in ER skills explained part of the treatment effect variance, as substantiated by a significant indirect effect. Accordingly, the interpretations that were derived regarding the first two hypotheses can be further substantiated by these findings, as they offer more scientifically sound conclusions. These conclusions incorporate that the improvement in ER skills, induced by the training, accounts for the reduction in procrastination scores and thereby the observed treatment effect. These initial findings further strengthen the postulated prediction that the training itself enhances ER skills through the increase in adaptive ER skills, which subsequently allow adaptive coping with antecedent aversive emotions, ultimately aiding in the prevention of procrastination.

In line with the procrastination-health model (Sirois et al., 2003), procrastination bears the risk of causing poor health outcomes through a stress-related and a behavioral route. Adaptive ER is associated with decreased stress (Katana et al., 2019) and allows adaptive coping with aversive emotions instead of adducting to maladaptive impulsive behavioral coping strategies (Berking et al., 2012), such as procrastination or even eating unhealthy foods. Therefore, regarding the present findings, it can be assumed that procrastination interventions, enhancing the adaptive ER skills applied within this training manual, do not only significantly reduce subsequent procrastination, instead they additionally diminish and prevent the associated adverse health consequences of both the stress-related and behavioral route. In line with these assumptions, research has shown that aversive emotions do not only represent key antecedents of procrastination, but instead most often equally of various mental disorders (Liverant et al., 2008; Berking et al., 2011), given that they both rely on maladaptive ER processes (Berking and Stumpenhorst, 2012). Therefore, it becomes evident why chronic procrastination exerts such a strong negative influence on students’ mental health and well-being through, e.g., increased levels of stress (Rice et al., 2012) or symptoms of depression (Essau et al., 2008; Flett et al., 2016). This research has shown that the enhancement of adaptive ER skills hinders aversive emotions from causing subsequent procrastination. Moreover, since maladaptive ER and the lack of adaptive ER skills is associated with many mental disorders, the acquisition of adaptive ER skills is suspected to additionally prevent the emergence of the associated adverse health effects of procrastination as well as other mental disorders based on impaired ER (Wirtz et al., 2013; Berking et al., 2014; Cloitre et al., 2019). Recent findings have already demonstrated that increasing ER skills can cause a reduction in depression (Eckert and Tarnowski, 2017; Berking et al., 2019) or improve the effect of cognitive behavioral therapy interventions for major depression (Berking et al., 2013). Especially taking into account that 75% of mental health issues are developed by the age of 25 and show an increase over the last decades (McManus et al., 2016), ER trainings should be considered a necessary intervention to diminish procrastination, its corresponding negative health consequences and prevent additional mental health issues.

Therefore, as a practical implication of these findings, educational institutions, especially universities, are encouraged to implement online-based trainings to enhance general ER skills. These trainings could allow a reduction in procrastination, symptoms of depression and an increase in well-being (Eckert and Tarnowski, 2017) and mental health (Berking et al., 2014). Especially, taking into account that the applied online ER training represents a cost-efficient and effective measure to reduce procrastination students should be provided with such trainings. The APOLLON University of Applied Sciences in Bremen in Germany already provides the German online version of the ER training “Stark im Stress” for all students for free and an English version is planned.

Thus, as students represent the social group that procrastinates most (Steel, 2007) and have a high risk for developing mental health issues (McManus et al., 2016), targeting ER skills to reduce procrastination, alleviate the respective health issues, and even promote buffering adaptive skills could provide a major step toward a healthier and more productive student population.

Taken together, the present findings highlight that the applied training of general ER skills (a) provided statistically significant increases in general adaptive ER skills, (b) significantly reduced the magnitude of subsequent procrastination behavior among the treated IG compared to the untreated WLC, which (c) was mediated by the overall increase in total ER skills. Adding onto preliminary findings, the present results replicate, albeit in part, the previous findings of Eckert et al. (2016) and beyond add insights on a more universal construct level. Conclusively, the value and major contribution of this study constitute that the present findings reveal unique first evidence indicating that the sole enhancement of general, non-task-specific ER skills significantly reduces subsequent procrastination. Therefore, these findings are highly valuable for current scientific literature, as they offer a more directed foundation for future research and for practical interventions aiming at diminishing the adverse effects of procrastination.

### Limitations

Despite the scientific and practical value of this research, the present study holds certain limitations. These limitations ought to be taken into consideration when interpreting the present results and initiating further research.

As this study has a longitudinal design with two measurements, covariables might have influenced the present findings and lowered the internal validity, such as changes within participants due to maturation, historical events, and learning effects. Regarding historical events, especially the ongoing COVID-19 pandemic must be mentioned. However, as (a) both groups were equally affected by the circumstances and accordingly, potential confounds are equally distributed and (b) the training was initiated during the lockdown period, which might even have reduced potential confounds, as usual external factors were kept to a minimum, these limitations can be considered negligible. Additionally, the study is limited to two measurements, precluding predictions about effect stability. However, as a first step, this study revealed that enhancing general ER skills decreases procrastination. Nonetheless, future research is needed to investigate long-term effects. Another possible source of error constitutes the large number of psychology students within the present sample. Considering that psychology students are, on average, more conscientious and achievement-oriented, they are expected to generally procrastinate less, which might have affected the present results. However, the present sample can be considered rather diverse, given that approximately 50% of the sample consisted of students of other faculties (economics and law, STEM fields, cultural, media and communication, and social sciences), as most psychological research is conducted exclusively on psychology students (Smart, 1966). The imbalanced gender ratio, with 75% female participants, however, does provide a limitation, especially as several studies have indicated that procrastination appears to be more represented among male students (e.g., Prohaska et al., 2000; Balkis and Duru, 2009; Kahn et al., 2014). The homogenous student sample does, on the one hand, increase the internal validity, however, limits the external validity. Therefore, generalizing predictions about other populations should be considered with caution, as generalizations from students to the general public can bear difficulties (Hanel and Vione, 2016). Additionally, self-selection biases (Lavrakas, 2008) might have affected the present results, as participants voluntarily signed up for this study. The non-existence of blinding of participants could account for further biases and could have functioned as placebo since participants were aware of the research object (the alleviation of procrastination through the training). Unfortunately, this study did not include an active control group with the administration of a placebo. Accordingly, effects due to placebo cannot be excluded. Another potential source of error constitutes the self-report measurement instrument of the dependent variable (procrastination) and the mediator variable (emotion regulation skills), which might encompass response biases (Del Boca and Noll, 2000). Nonetheless, it must be acknowledged that both questionnaires are validated and represent convergent and discriminant valid measures of the examined constructs (GPS-K; Klingsieck and Fries, 2012; ERSQ; Berking and Znoj, 2008). Furthermore, it should be noted that the randomization was only partially successful as there were significant distribution differences regarding gender and course of studies between the treatments. However, as the largest groups (psychology and business students) were distributed evenly, it can be assumed that the course of study allocation difference had no distorting influence on the results. Moreover, as they were due to randomization, they can be considered negligible.

### Future Research

As already indicated in the section “Limitations,” further research is necessary to substantiate the present findings and overcome the appeared restrictions. First, considering the scarce prior research within the field of ER in relation to procrastination, further replicating scientific work is needed to confirm and evaluate the detected treatment and mediation effects. Especially, replicative RCTs with active control groups should be considered to preclude that the findings confounded with a placebo. The present results are encouraging yet should be replicated with other samples, especially representative adult samples, to ensure the practical value of ER interventions in the prevention of procrastination beyond the educational context. Effects among adult populations would allow a transfer of the present implications onto a different population, and therefore, the opportunity to reduce procrastination within a broader target group at a more generalizing population level. Further research should investigate the effects of different ER skills trainings on procrastination to examine potential differences between trainings and to ensure the independence of the findings from the selected training program. The present results suggest that as maladaptive coping with preceding negative affect is reduced, and adaptive coping is enhanced by improving adaptive ER skills, subsequent procrastination diminishes. Since maladaptive coping mediates the relationship between procrastination and its associated negative health consequences (e.g., Sirois and Tosti, 2012; Sirois and Kitner, 2015), further research should validate the prediction that the enhancement of ER skills permits a reduction of procrastination’s related negative health consequences through the enhancement of adaptive coping. It follows logically that future research should investigate adaptive coping and potential improvements in subsequent health indicators, to test whether the improvements in the procrastination-related impaired health indicators are attributable toward the increases in adaptive coping through the enhancement of adaptive ER skills. Additionally, alterations in performance indicators such as course grades or work productivity should longitudinally be assessed to further evaluate the practical value of ER training interventions. Conclusively, the present study provides a seminal base for future studies on the prevention of procrastination.

## Conclusion

Taken together, considerable progress has been made with the present study. The present findings add onto a scarce yet growing body of literature supporting previous assumptions about the interrelationship of ER skills and procrastination. Most strikingly, the present results provide first-time novel insights on a more universal construct level. This study has contributed toward enhancing our understanding of how the broad constructs of ER and procrastination are related to each other, showing that the exclusive enhancement of general adaptive ER skills significantly reduces procrastination behaviors. Moreover, this research precisely clarified through subsequent significant mediation analyses of ER skills which facets of the broad construct ought to be enhanced to significantly reduce procrastination behavior. Even though further empirical work needs to be initiated to replicate the present findings and overcome the limitations, the present results notably contribute to our current knowledge of procrastination as a dysfunctional ER strategy and depict an essential step toward overcoming the phenomenon.

## Data Availability Statement

The raw data supporting the conclusions of this article will be made available by the authors, without undue reservation.

## Ethics Statement

The studies involving human participants were reviewed and approved by the Leuphana University of Lüneburg. The patients/participants provided their written informed consent to participate in this study.

## Author Contributions

LS executed and administered the study, wrote the first version of the manuscript, and composed the final version in cooperation with ME. VS corrected and advised regarding stress regulation. MS corrected and advised regarding ER. ME developed the online training SIS, supported the development of the study design, and composed the final version of the manuscript in cooperation with LS.

## Conflict of Interest

The authors declare that the research was conducted in the absence of any commercial or financial relationships that could be construed as a potential conflict of interest.

## Publisher’s Note

All claims expressed in this article are solely those of the authors and do not necessarily represent those of their affiliated organizations, or those of the publisher, the editors and the reviewers. Any product that may be evaluated in this article, or claim that may be made by its manufacturer, is not guaranteed or endorsed by the publisher.

## Footnotes

1
https://leuphana.sona-systems.com/

2
https://www.training-sis.de
